# Oxygen consumption dynamics in steady-state tumour models

**DOI:** 10.1098/rsos.140080

**Published:** 2014-09-24

**Authors:** David Robert Grimes, Alexander G. Fletcher, Mike Partridge

**Affiliations:** 1 Cancer Research UK/MRC Oxford Institute for Radiation Oncology, Gray Laboratory, University of Oxford, Old Road Campus Research Building, Off Roosevelt Drive, Oxford OX3 7DQ, UK; 2 Wolfson Centre for Mathematical Biology, Mathematical Institute, University of Oxford, Andrew Wiles Building, Radcliffe Observatory Quarter, Woodstock Road, Oxford OX2 6GG, UK

**Keywords:** mathematical modelling, hypoxia, oxygen

## Abstract

Oxygen levels in cancerous tissue can have a significant effect on treatment response: hypoxic tissue is both more radioresistant and more chemoresistant than well-oxygenated tissue. While recent advances in medical imaging have facilitated real-time observation of macroscopic oxygenation, the underlying physics limits the resolution to the millimetre domain, whereas oxygen tension varies over a micrometre scale. If the distribution of oxygen in the tumour micro-environment can be accurately estimated, then the effect of potential dose escalation to these hypoxic regions could be better modelled, allowing more realistic simulation of biologically adaptive treatments. Reaction–diffusion models are commonly used for modelling oxygen dynamics, with a variety of functional forms assumed for the dependence of oxygen consumption rate (OCR) on cellular status and local oxygen availability. In this work, we examine reaction–diffusion models of oxygen consumption in spherically and cylindrically symmetric geometries. We consider two different descriptions of oxygen consumption: one in which the rate of consumption is constant and one in which it varies with oxygen tension in a hyperbolic manner. In each case, we derive analytic approximations to the steady-state oxygen distribution, which are shown to closely match the numerical solutions of the equations and accurately predict the extent to which oxygen can diffuse. The derived expressions relate the limit to which oxygen can diffuse into a tissue to the OCR of that tissue. We also demonstrate that differences between these functional forms are likely to be negligible within the range of literature estimates of the hyperbolic oxygen constant, suggesting that the constant consumption rate approximation suffices for modelling oxygen dynamics for most values of OCR. These approximations also allow the rapid identification of situations where hyperbolic consumption forms can result in significant differences from constant consumption rate models, and so can reduce the computational workload associated with numerical solutions, by estimating both the oxygen diffusion distances and resultant oxygen profile. Such analysis may be useful for parameter fitting in large imaging datasets and histological sections, and allows easy quantification of projected differences between functional forms of OCR.

## 2. Introduction

It has been known since the pioneering work of Gray and co-workers in the 1950s [1] that well-oxygenated tissue responds better to radiotherapy and chemotherapy than by up to a factor of 3 relative to tumours with extensive hypoxia. This relative boosting effect of oxygen on cell-kill is often quantified by the oxygen enhancement ratio (OER), which is of fundamental importance in radiotherapy [2]. The prognostic influence of hypoxia has led to the concept of dose painting [3,4] in radiotherapy, which proposes that hypoxic regions of a tumour could be given an increased dose to mitigate their inherent radioresistance. Imaging modalities such as positron emission tomography (PET) can be coupled with hypoxia binding tracers such as 18F-fluoromisonidazole to allow the non-invasive estimation of hypoxia *in vivo* [3]. Although this approach is promising, the spatial resolution remains prohibitively low [5], as PET imaging is physically constrained to a millimetre-scale resolution, while oxygen tension is known to vary significantly over tens of micrometres [6–9], owing to the relatively short oxygen diffusion distances in tissue.

To maximize the potential advantages of a dose painting approach to treatment outcome, it is vital that the underlying oxygen distributions are well understood. Mathematical modelling of oxygen transport at micrometre scales is therefore required to estimate oxygen distributions. In continuum formulations, oxygen is assumed to diffuse through tissue while simultaneously being consumed by the respiring cells. Models of this type have been developed for spherical geometries to describe the oxygen tension in multicellular tumour spheroid (MCTS) growth [10–12] and cylindrical geometries for blood vessel perfusion modelling [8,9,13,14]. This approach has been widely used in various tissue geometries and situations [8–11,15–20] to estimate the oxygen dynamics through a tissue. Several functional forms of the oxygen consumption rate (OCR) have been proposed. In some formulations, it is treated as a constant [8,9] while elsewhere the OCR is assumed to vary with oxygen tension, typically obeying a rectangular hyperbolic relationship, similar in form to Michaelis–Menten kinetics or the Langmuir adsorption isotherm. This particular functional form has been shown to be a good approximation in a variety of tissues, both healthy and cancerous [21–24]. A typical hyperbolic reaction rate is given by

2.1 $$\frac{\nu_{\max}s}{s+K_{m}},$$

where *s* is the substrate concentration, $\nu_{\max}$ is the maximum reaction rate and *K*_*m*_ is substrate level where *ν* is half $\nu_{\max}$.

For cellular oxygen consumption, this model is phenomenological, and its sigmoidal form is intended to capture the observation that the OCR in a given tissue is typically lower under hypoxic conditions than under normoxia. From a biological perspective, such dynamics may be explained by the observation that under hypoxic conditions, expression of proteins such as HIF-1 can lead to downregulation of mitochondrial oxygen consumption, as well as affecting cell cycling [25]. The nonlinear form of (1.1) renders reaction–diffusion equations involving hyperbolic kinetics analytically intractable in general, and so they must be solved numerically.

The form of equation (1.1) suggests that the relative magnitude of the constant *K*_*m*_ for oxygen has a distinct effect on the shape of the consumption rate curve. The literature to date indicates that this constant for oxygen is quite low compared with typical oxygen tension in a vessel, typically *K*_*m*_≃1 mm Hg [26–28]. This may suggest only small predicted differences between the two consumption rate forms described above in most cases, though there is no simple method for estimating which combinations of parameters may result in a significant difference between both forms. More importantly perhaps, the oxygen diffusion limit could differ significantly between both models; consider two reaction–diffusion equations with a maximum OCR of *a*, the first operating under the assumption of constant OCR and the second under the assumption of hyperbolic kinetics. For the first model, OCR is intrinsic and is not modulated by oxygen availability so oxygen consumption is *a* throughout. In the second model, the OCR is modulated by the presence of oxygen and is given by *aP*/(*P*+*K*_*M*_), where *P* is the local oxygen partial pressure. In this case, oxygen consumption will reduce with local oxygen availability, allowing oxygen to diffuse deeper into the respiring tissue than in the constant case. Thus, if all other parameters are kept the same, the oxygen diffusion distance in the hyperbolic model will be always greater than for the constant consumption rate case. Under what circumstances this difference may be significant in a given situation is an open question, and part of the motivation for this work.

Previous literature has outlined how oxygen diffusion distance can be analytically derived for the constant consumption rate case in the case of cylindrical [8] and spherical [10] geometries, and to date numerical approaches have been used when considering non-constant kinetics, owing to the analytic intractability of the problem [29]. The significant drawback of such approaches is that the diffusion boundaries must be specified by the user, rendering the process uncertain and making direct comparison between constant oxygen consumption models and hyperbolic kinetic models difficult. Despite the small literature estimates of *K*_*m*_ for oxygen, there may be cases where significant differences emerge between constant consumption and M–M case. It would be beneficial to have a simple method of quantifying the effects of both constant OCR and hyperbolic consumption rate forms, and a specifically a method for estimating the predicted oxygen diffusion distance in the hyperbolic case explicitly. Such a method would also have to be capable of explicitly relating OCR to the oxygen diffusion distance. In this work, we derive analytical approximations for hyperbolic-type models in both spherical and cylindrical geometry, and contrast these to constant consumption models. We show how oxygen diffusion distance is governed by OCR, and we show how the models derived can be used to obtain a value for the oxygen diffusion distance and rapid estimation of the expected oxygen profile. As the oxygen tension and associated boundaries can be estimated analytically, this can reduce computational workload when modelling systems of such elements or performing image analysis on vessel sections. We present results of this analysis for key quantities of interest such as expected diffusion distance and oxygen distribution and discuss the implications for oxygen modelling at a micrometre scale.

## 3. Methods and models

### 3.1 Spherical symmetry

We adopt a continuum modelling approach, deriving a reaction–diffusion equation governing the spatio-temporal dynamics of oxygen in an avascular tissue. Adopting spherical polar coordinates, we suppose that the centre of the MCTS is located at *r*=0. Since the average doubling time of cells in an MCTS is much longer than the typical time scale of oxygen diffusion across the spheroid, we make the simplifying assumption that the spheroid radius, denoted by *r*_*o*_, is constant over the time scale of measurement.

We initially suppose that the spheroid is sufficiently large that a central anoxic region exists where the oxygen concentration is zero. We denote the radius of this anoxic region by *r*_*n*_, so that oxygen partial pressure is zero in the region 0≤*r*≤*r*_*n*_, and *r*_*n*_ is the boundary to which oxygen can penetrate from *r*_*o*_. For convenience, we let *r*_*c*_=*r*_*o*_−*r*_*n*_ denote the width of the viable rim of the tissue. We further assume the MTCS is suspended in a well-mixed growth medium [10,30].

**Figure 1.**
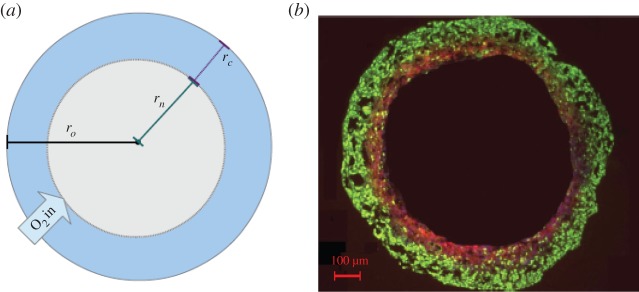
(*a*) Spherical geometry: a cross section of a tumour spheroid of radius *r*_*o*_. Oxygen partial pressure at *r*_*o*_ is *p*_*o*_ and oxygen partial pressure falls to 0 at *r*_*n*_, the radius of the anoxic region. (*b*) A cross section of a DLD-1 colorectal spheroid with same regions visible [10].

We assume that spherical symmetry is preserved, as illustrated in figure 1, and we let *p*(*r*,*t*) denote the partial pressure of oxygen at a distance *r* from the centre of the MCTS at time *t*. We consider changes in oxygen partial pressure due to two processes: Fickian diffusion into and within the MCTS, and cellular consumption. We also make the simplifying assumption that the diffusion coefficient of oxygen, denoted by *D*, is spatially invariant. We let *a*(*p*) denote the rate of cellular consumption of oxygen, which may depend on the local oxygen partial pressure. It can readily be shown that for typical literature values of the oxygen diffusion constant *D* that oxygen diffusion through an MCTS occurs on a time scale of seconds and a steady-state approximation is therefore acceptable, under which *p* satisfies the reaction–diffusion equation

3.1 $$0=\frac{D}{r^{2}}\frac{d}{dr}\left(r^{2}\frac{dp}{dr}\right)-\Omega a(p)$$

in the region *r*_*n*_<*r*<*r*_*o*_, where *Ω*=3.0318×10^7^ mm Hg kg m^−3^ arises from Henry’s law as outlined in previous work [10]. Although one could readily define a constant that subsumes *a*(*p*) and *Ω*, we will keep them separate in this work to reflect their physical meaning—specifically *a*(*p*) is the oxygen consumption rate in SI units of volume of oxygen per unit mass per unit time. If the two terms are subsumed into a resultant term *Ωa*(*p*), then this yields consumption in units of oxygen partial pressure per unit time. To close this system, we must impose appropriate boundary conditions. We impose the regularity condition *p*(*r*_*n*_)=0 at the anoxic edge and assume that the exterior medium is well mixed and that oxygen is in excess there. This is equivalent to assuming a very high mass transfer coefficient for *p* at *r*=*r*_*o*_ leading to the Dirichlet boundary condition *p*(*r*_*o*_)=*p*_*o*_. It is important to note that *r*_*n*_, the anoxic radius, is not known *a priori* but can be derived from first principles, as outlined below.

#### 3.1.1 Constant consumption rate

If the OCR is taken to be constant then it is possible to derive a closed-form analytic solution to the model [10]. In this case, oxygen consumption is a constant so that *a*(*p*)=*a*_*o*_. The exact solution is given analytically by

3.2 $$p(r)=p_{o}+\frac{a_{o}\Omega }{6D}\left(r^{2}-r_{o}^{2}+2r_{n}^{3}\left(\frac{1}{r}-\frac{1}{r_{o}}\right)\right)=\frac{a_{o}\Omega }{6D}\left(r^{2}+\frac{2r_{n}^{3}}{r}-3r_{n}^{2}\right)$$

for *r*_*n*_≤*r*≤*r*_*o*_. It can further be shown [10] that the maximum radius that oxygen can penetrate before the formation of an anoxic core is given by

3.3 $$r_{l}=\sqrt{\frac{6Dp_{o}}{\Omega a_{o}}}.$$

It is important to note that *r*_*n*_ is not known *a priori* and needs to be calculated. In equation (2.2), the anoxic radius *r*_*n*_ is undetermined and can be explicitly calculated [10] to give

3.4 $$r_{n}=r_{o}\left(\frac{1}{2}-\cos\left(\frac{\arccos(1-2r_{l}^{2}/r_{o}^{2})-2\pi }{3}\right)\right)\;.$$

For spheroids sufficiently small to have no central anoxia, *r*_*n*_=0 and a similar analysis can be performed.

#### 3.1.2 Hyperbolic consumption rate

In the hyperbolic case, there is no exact analytical solution to equation (2.1). The general approach taken in the literature has been to use numerical methods to estimate the local partial pressure [11]. A drawback of this approach is the lack of a closed-form expression for the boundary of the anoxic radius, *r*_*n*_. This differs from that in the constant model, as the reduced OCR in the hyperbolic formulation yields a longer diffusion distance. Denoting this modified anoxic radial distance by $r_{n}^{*}$, we can establish a phenomenological approximation which captures the behaviour of the function and satisfies the conditions that *a*(*p*)=0 at the anoxic edge *r*_*n*_ with a maximum value for *a*(*p*) at *r*_*o*_. Close to the interface between oxygenated and hypoxic regions, the oxygen curve falls to zero with a zero gradient, which can be readily approximated by a quadratic parabola-like fit. If we define *a*_*o*_ as the maximum OCR, then a suitable approximation for *a*(*p*) is given by

3.5 $$a(p)=\frac{a_{o}p(r)}{p(r)+K_{m}}\approx \frac{a_{o}{\left(r-r_{n}^{*}\right)}^{2}}{{\left(r-r_{n}^{*}\right)}^{2}+k_{r}},$$

where

3.6 $$k_{r}={\left(r_{o}-r_{n}^{*}\right)}^{2}\frac{K_{m}}{p_{o}}.$$

Substituting (2.5) into (2.1) with the same boundary conditions as the constant consumption rate case, $p(r_{n}^{*})=0$ and $p^{\prime }(r_{n}^{*})=0$, we obtain the analytic solution

3.7 $$\begin{array}{rl} p(r) & =\frac{a_{o}\Omega }{6D}(r^{2}-3{r_{n}^{*}}^{2}+\frac{2{r_{n}^{*}}^{3}-6k_{r}r_{n}^{*}}{r}+6k_{r}-3\sqrt{k_{r}}\pi r_{n}^{*}+6\sqrt{k_{r}}r_{n}^{*}\arctan\left(\frac{\sqrt{k_{r}}}{r-r_{n}^{*}}\right)\\ & \;+\frac{\left(6\sqrt{k_{r}}{r_{n}^{*}}^{2}-6k_{r}^{3/2})\arctan\;((r-r_{n}^{*})/\sqrt{k_{r}}\right)}{r}+\left(\frac{6k_{r}r_{n}^{*}}{r}-3k_{r}\right)\ln\left(\frac{{\left(r-r_{n}^{*}\right)}^{2}+k_{r}}{k_{r}}\right))\;. \end{array}$$

To estimate $r_{n}^{*}$ in this case, (2.8) is solved subject to *p*(*r*_*o*_)=*p*_*o*_. From this, $r_{n}^{*}$ can be found to any required degree of precision from the resulting expression, allowing determination of this boundary. It is important to note that oxygen can diffuse further in the hyperbolic formulation, and subsequently the anoxic region is decreased relative to the constant consumption case so that $r_{n}^{*}\leq r_{n}$. In the case of spheroids sufficiently small to have no central anoxia, $r_{n}^{*}=0$.

### 3.2 Cylindrical symmetry

**Figure 2.**
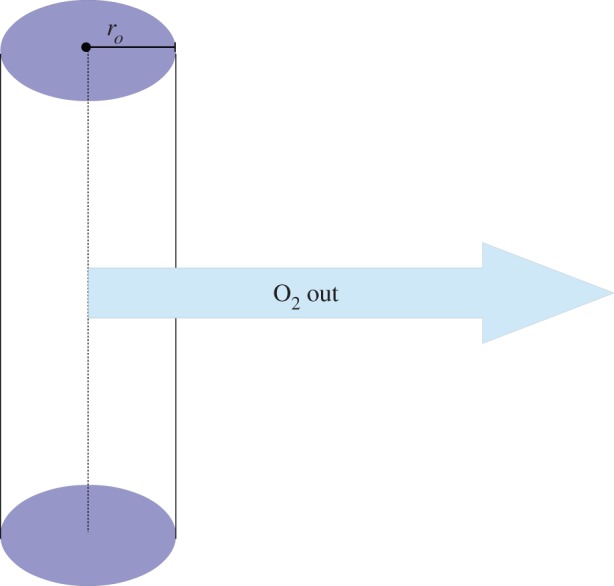
Schematic diagram of a Krogh-type vessel model in cylindrical geometry, with vessel radius *r*_*o*_.

Cylindrically symmetric reaction–diffusion equations are often encountered in Krogh-type models to simulate oxygen flow from a blood vessel [8,9,13] under the reasonable assumption that vessels secrete oxygen perpendicular to their walls. In these situations, they present a reasonable approximation of a length of blood vessel running through a tumour. Such a situation is illustrated in figure 2. In this case, the steady-state oxygen distribution satisfies

3.8 $$0=\frac{D}{r}\frac{d}{dr}\left(r\frac{dp}{dr}\right)-a(p)\Omega$$

for all 0≤*r*≤*r*_*c*_, where *r*_*c*_ is the diffusion limit from the vessel.

#### 3.2.1 Constant consumption rate

With a constant consumption rate, *a*_*o*_, an exact solution is possible. For a vessel of radius *r*_*o*_ with boundary conditions *p*(*r*_*c*_)=*p*′(*r*_*c*_)=0 and *p*(*r*_*o*_)=*p*_*o*_, the solution to (2.8) is given by

3.9 $$p(r)=p_{o}+\frac{a_{o}\Omega }{4D}\left(r^{2}-r_{o}^{2}-2r_{c}^{2}\ln\frac{r}{r_{o}}\right)\;.$$

Unlike the spherically symmetric case, there is no corresponding analytical expression for the diffusion distance *r*_*c*_ but this can be readily estimated using root-finding methods when equation (2.9) is equal to zero.

#### 3.2.2 Hyperbolic consumption rate

Analysis analogous to the spherical geometry can be applied in the hyperbolic case, with the caveat that the substitution term is modified, as in this case diffusion is out from the vessel and must be zero at the radius $r_{c}^{*}$. In this case, oxygen should diffuse further from the vessel than in the constant consumption rate case. This approximation is given by

3.10 $$a(p)=\frac{a_{o}p(r)}{p(r)+K_{m}}≃\frac{a_{o}{\left(r_{c}^{*}-r\right)}^{2}}{{\left(r_{c}^{*}-r\right)}^{2}+l_{r}},$$

where

3.11 $$l_{r}={\left(r_{c}^{*}-r_{o}\right)}^{2}\frac{K_{m}}{p_{o}}.$$

Solving (2.8) subject to the boundary conditions $p(r_{c}^{*})=p^{\prime }(r_{c}^{*})=0$ and *p*(*r*_*o*_)=*p*_*o*_ yields an expression that is analytically tractable, but unwieldy. The first derivative of the solution is given by

3.12 $$p^{\prime }(r)=\frac{a_{o}\Omega }{2D}\left(r+\frac{2r_{c}^{*}\sqrt{l_{r}}\arctan\;((r_{c}^{*}-r)/\sqrt{l_{r}})-l_{r}\ln({\left(r_{c}^{*}-r\right)}^{2}/l_{r})-{r_{c}^{*}}^{2}}{r}\right)\;.$$

To find $r_{c}^{*}$, the modified diffusion distance, equation (2.12) is integrated with respect to *r* subject to $p(r_{c}^{*})=0$. Equation (2.12) can be solved analytically, producing a long expression which is not shown here for the sake of brevity. The full solution and code is supplied in the electronic supplementary material for reference. The resulting form is then evaluated with condition *p*(*r*_*o*_)=*p*_*o*_ so that $r_{c}^{*}$ may be found to any desired degree of accuracy.

### 3.3 Simulations and comparison with numerical solutions

In both geometries, the oxygen distributions resulting from both models were simulated with the maximum consumption rate parameter *a* varying over an order of magnitude so that 2.5×10^−7^≤*a*≤1.5×10^−6^ m^3^ kg^−1^ s^−1^, corresponding to the range 7.50–45.45 mm Hg *O*_2_ s^−1^. For the spherical geometry, the external partial pressure was taken as 100 mm Hg. In the cylindrical geometry, both ‘arterial’ (100 mm Hg) and ‘venous’ (40 mm Hg) source partial pressures were considered and vessel radius was fixed at *r*_*o*_=5 μm. These values were chosen as illustrations of the typical case, but in tumours chaotic vasculature often means significant variation in these values and can result in lower oxygen partial pressures [31], so for the cylindrical case a chaotic partial pressure of 20 mm Hg was also simulated. A value for the diffusion constant close to that of water (*D*=2×10^−9^ m^2^ s^−1^) [8,9] was also used.

For each set of simulation parameters, the oxygen tension profile and diffusion length (anoxic radius in the spherical geometry, diffusion distance in the cylindrical geometry) were compared and the differences between both models for the given parameter set computed. The analytical approximations were contrasted with the numerical solutions to gauge how well they described the underlying behaviour of these models. As the value of *K*_*m*_ in the literature is typically ≤1 mm Hg [24,26], a value of 1 mm Hg was used in these simulations. To examine the sensitivity of the model to this parameter, simulations using values of 0 mm Hg≤*K*_*m*_≤10 mm Hg were examined.

## 4. Results

### 4.1 Validity of the analytical approximation

**Figure 3.**
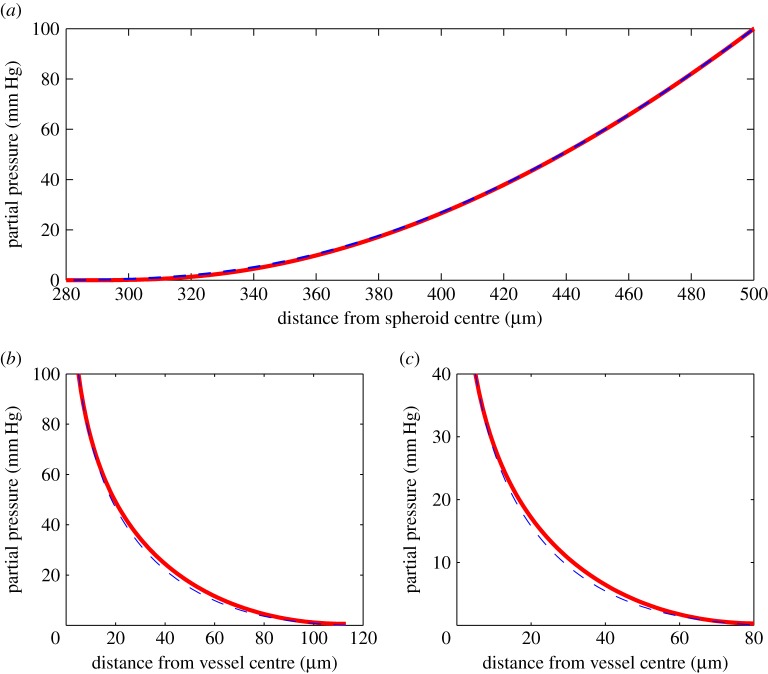
Comparisons of the approximation solution (solid line) with numerical solution (dashed line) for (*a*) a 500 μm spheroid ($r_{n}^{*}=281.62\;\mu m$ and *p*_*o*_= 100 mm Hg), (*b*) a vessel of radius *r*_*o*_=5 μm ($r_{c}^{*}=113.12\;\mu m$, *p*_*o*_= 100 mm Hg, typical arterial pressure) and (*c*) a vessel of radius *r*_*o*_=5 μm ($r_{c}^{*}=81.36\;\mu m$, *p*_*o*_=40 mm Hg, typical venous pressure). For examples here, *a*=5×10^−7^ m^3^ kg^−1^ s^−1^ and *D*= 2×10^−9^ m^2^ s^−1^.

**Figure 4.**
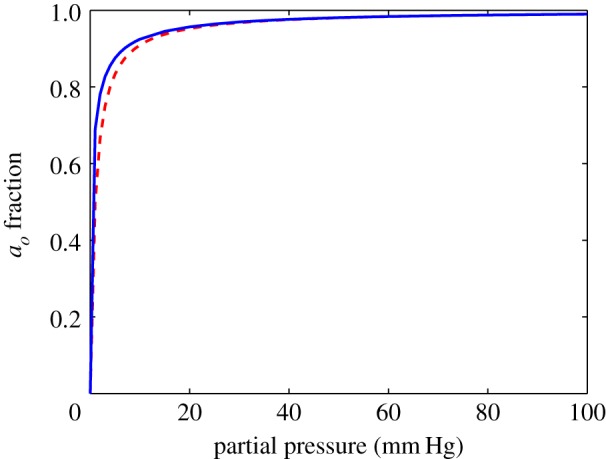
Comparison of the variation of relative oxygen consumption with oxygen partial pressure for the hyperbolic (dashed line) form (*P*/(*P*+*K*_*m*_)) and the approximation (solid line) derived in this work, outlined in equation (2.5).

To estimate the validity of our analytical approximation, we compared results from employing the approximation functions in both geometries with simulations using boundaries estimated from the approximation functions. Typical comparisons are shown in figure 3. In spherical geometry, the agreement is exceptionally good. Differences between the numerical simulation and analytical approximation were almost negligible, with only small disagreement at very low oxygen partial pressures (Δ*p*<0.24 mm Hg), indicating that the spherical approximation describes the solution to equation (2.1) well (all errors ≪1 mm Hg) and yields the behaviour expected from the equation. The agreement of the hyperbolic function with the approximation outlined in equation (2.5) is shown in figure 4.

In the cylindrical case, the analytical approximation systematically overestimated the oxygen partial pressure by a slight amount for each simulated case as can be seen in figure 3. This may be due to the two-term quadratic truncation introducing an error. The mean error was typically 0.3±1 mm Hg. Despite this systematic slight overestimation, the profile agreement is still high and the functional behaviour consistent. This indicates that the analytical approximation describes the function behaviour well in both geometries.

### 4.2 Comparison of constant and hyperbolic consumption rate models

**Figure 5.**
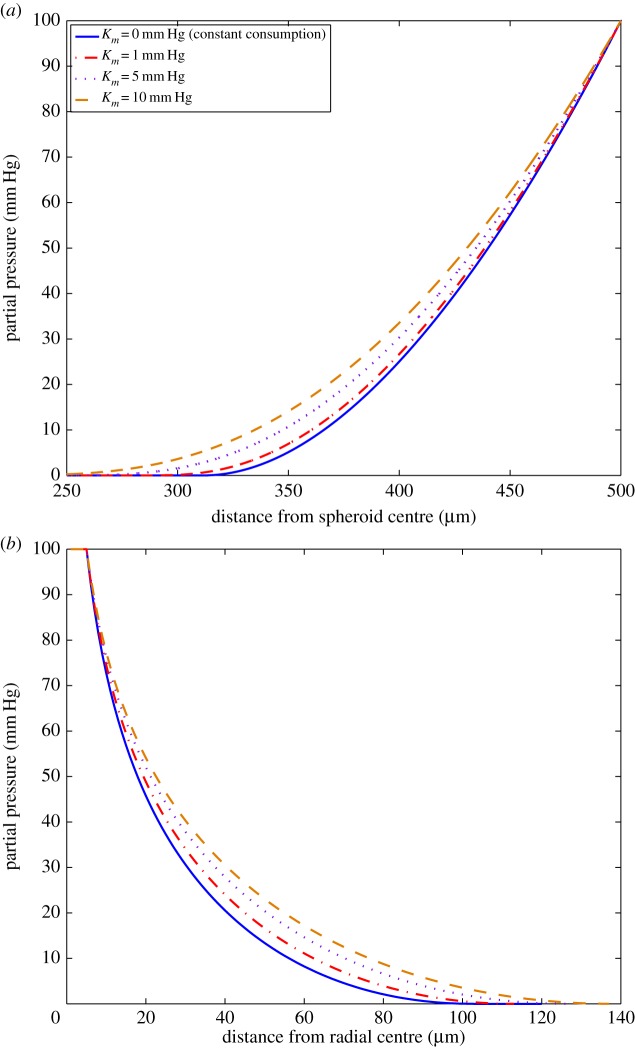
Comparing the constant and Michaelis–Menten consumption rate models for (*a*) spherical geometry (*r*_*o*_=500 μm) and (*b*) cylindrical geometry (*r*_*o*_=5 μm). In both cases, *D*=2×10^−9^ m^2^ s^−1^ and *a*=5×10^−7^ m^3^ kg^−1^ s^−1^. When *K*_*m*_=0 mm Hg, the hyperbolic model reduces to the constant consumption case. Increased values of *K*_*m*_ result in longer oxygen diffusion distances and higher overall oxygen profiles.

Table 1 shows the estimated difference in boundary location between both models for a spherical geometry in the domain $r_{n}^{*}\leq r\leq r_{o}$. In all cases, oxygen penetrates deeper into the spheroid in the hyperbolic situation, with these models having a decreased anoxic radius relative to the constant consumption model. Typically, this difference, Δ*r*_*n*_, is of the order of tens of micrometres as shown in table 1. Despite the increased oxygen penetration and reduced anoxic radius in the hyperbolic model however, the oxygen profile is largely consistent between both models for spherical geometry. An example of this is illustrated in figure 5*a*, where the average difference between both profiles is only 0.35±0.60 mm Hg in the region $r_{n}^{*}\leq r\leq r_{o}$. Even in the relatively small region of interest $r_{n}^{*}\leq r\leq r_{n}$, with greatest possible differences between both models, the mean difference is only 1.01±0.61 mm Hg. When the entire spheroid (0≤ *r*≤*r*_*o*_) is considered, differences between both models are effectively negligible, being typically <0.5 mm Hg.

**Table 1.** Comparison of anoxic radii (*r*_*n*_ and $r_{n}^{*}$) and average difference in oxygen partial pressure $\left(\bar{\Delta }p\right)$ for constant and hyperbolic consumption rate models in spherical geometry.

*a* (m^3^ kg^−1^ s^−1^)	*r*_*o*_ (μm)	*r*_*n*_ (μm)	$r_{n}^{*}$ (μm)	Δ*r*_*n*_ (μm)	$\bar{\Delta }p$ (mm Hg)
2.5×10^−7^	500	204	153	51	0.85±0.86
	750	487	445	42	0.42±0.65
	1000	747	708	40	0.29±0.55
5×10^−7^	500	312	282	30	0.46±0.67
	750	572	545	28	0.27±0.53
	1000	827	800	27	0.19±0.45
7.5×10^−7^	500	351	328	23	0.35±0.60
	750	608	586	22	0.21±0.47
	1000	860	839	22	0.15±0.40
1×10^−6^	500	374	354	20	0.29±0.55
	750	628	609	19	0.17±0.43
	1000	880	861	19	0.13±0.37
1.25×10^−6^	500	388	371	17	0.25±0.51
	750	642	625	17	0.15±0.41
	1000	893	877	17	0.11±0.35
1.5×10^−6^	500	399	383	16	0.22±0.49
	750	652	636	15	0.14±0.39
	1000	903	888	15	0.10±0.33

Similar trends are seen in the case of a cylindrical geometry, as shown in table 2. For the typical example illustrated in figure 5*b*, the average difference between the two models was 2.17±1.25 mm Hg over the entire profile, and a mean difference of 0.13±0.12 mm Hg in the region $r_{c}\leq r\leq r_{c}^{*}$. For all simulated cylindrical profiles, the difference in this region is negligible. The difference in diffusion limit Δ*r*_*c*_ is relatively small in this geometry at around 10 μm. However, over the entire region 0≤*r*≤*r*_*c*_, differences between the constant consumption and hyperbolic consumption rate models are more pronounced in the cylindrical geometry.

**Table 2.** Comparison of diffusion-limited radii (*r*_*c*_ and $r_{c}^{*}$) and average difference in oxygen partial pressure ($\bar{\Delta }p$) for constant and hyperbolic consumption rate models in cylindrical geometry (*r*_*o*_=5 μm).

*p*_*o*_ (mm Hg)	*a* (m^3^ kg^−1^ s^−1^)	*r*_*c*_ (μm)	$r_{c}^{*}$ (μm)	Δ*r*_*c*_ (μm)	$\bar{\Delta }p$ (mm Hg)
100 (arterial pressure)	2.5×10^−7^	137	152	15	2.11±1.17
	5×10^−7^	102	113	11	2.14±1.23
	7.5×10^−7^	86	95	9	2.17±1.25
	1×10^−6^	77	85	8	2.18±1.27
	1.25×10^−6^	70	77	7	2.18±1.30
	1.5×10^−6^	65	72	6	2.19±1.30
40 (venous pressure)	2.5×10^−7^	93	109	15	1.20±0.77
	5×10^−7^	70	81	11	1.31±0.80
	7.5×10^−7^	60	69	9	1.32±0.82
	1×10^−6^	53	61	8	1.32±0.84
	1.25×10^−6^	49	56	7	1.33±0.84
	1.5×10^−6^	45	52	7	1.31±0.86
20 (chaotic pressure)	2.5×10^−7^	70	86	16	0.84±0.56
	5×10^−7^	53	64	11	0.89±0.58
	7.5×10^−7^	45	55	10	0.89±0.59
	1×10^−6^	41	49	8	0.90±0.60
	1.25×10^−6^	37	45	8	0.89±0.61
	1.5×10^−6^	35	42	7	0.89±0.62

Simulations of the hyperbolic model in both geometries were run with various values of *K*_*m*_ to investigate what effect this had on the expected oxygen profile. When *K*_*m*_=0 mm Hg, the hyperbolic model reduces to the constant consumption case as expected. For higher values of *K*_*m*_, the divergence between the constant and hyperbolic consumption cases increases with oxygen penetrating deeper and an increased overall oxygen profile. This behaviour is shown in figure 5 with *a*=5×10^−7^ m^3^ kg^−1^ s^−1^. At *K*_*m*_=10 mm Hg, an order of magnitude above literature values, oxygen diffuses approximately 55% further through the spheroid than in the constant consumption case. In the cylindrical case at *K*_*m*_=10 mm Hg, the diffusion distance is 30% greater than in the constant consumption case. This significantly changes the oxygen profile in both geometries as well as the diffusion distance with markedly different values expected for different values of *K*_*m*_. However, as literature values to date indicate that *K*_*m*_<1 mm Hg, this would suggest that the expected oxygen profile in all geometries should be very close to that of the constant consumption rate case. Consequently, it is unlikely that this issue would ever be a significant factor when oxygen is the substrate but may be an issue for applying these dynamics to substances other than oxygen.

## 5. Discussion

While the differences in oxygen profile between the constant and hyperbolic consumption rate models are relatively small, the increased oxygen diffusion distance in the hyperbolic case may have an impact when estimating the extent of the hypoxic region. In the regions of difference between the constant and hyperbolic rate models ($r_{n}^{*}\leq r_{n}$ in the spherical case and $r_{c}\leq r_{c}^{*}$ in the cylindrical case), oxygen tension is typically extremely low (≤1 mm Hg). This may have radiobiological significance, since OER falls to unity under tensions below 2.5 mm Hg, rendering such areas of tumour more radioresistant than the adjacent well-oxygenated regions [5]. However, the oxygen profile between the two models diverges only at very low oxygen tension, where both have increased radioresistance and should as a consequence have practically identical radio-response in most situations.

In spherical geometry, the divergence between the constant and hyperbolic consumption model is generally relatively minor, as can be seen in table 1. There are however situations where this is not the case; for example, spheroids with low consumption rates and a radius of less than 500 *μ*m, such as the first entry in table 1 for a spheroid with radius 500 μm and OCR of 2.5×10^−7^ m^3^ kg^−1^ s^−1^. In this case, the difference between *r*_*n*_ and $r_{n}^{*}$ can exceed 25%, and similarly parameter combinations exist that can yield such differences between the models. Even in these cases, the oxygen profiles for both formulations are broadly similar, differing only by very small values (≪1 mm Hg) in the domain $r_{n}^{*}<r<r_{n}$. In the spherical case, projected differences decrease markedly for higher consumption rates and larger spheroid radii. Although the model predictions between both constant and hyperbolic consumption forms tend to yield similar oxygen profiles for realistic estimates of *K*_*m*_, it might be possible to use stained spheroids to investigate this further. One option would be spheroids stained with EF5, which binds to oxygenated regions between 0.8 and 10 mm Hg [32]. This would manifest in small differences between the expected location of the stained boundary in both models, maximal at *p*≃0.8 mm Hg. In both spherical and cylindrical geometries, the projected differences are typically ≈10μm, which could allow discrimination between the models if the effective average consumption rate was accurately known. Although this could, in principle, be tested on stained spheroid sections, a difficulty arises due to the fact that the projected difference in staining boundary between the constant and hyperbolic consumption models for the same maximal consumption rate *a* is of the same order of magnitude as the uncertainties in estimating the stained boundary [10], rendering direct comparison extremely difficult. Conversely, this implies that at current experimental resolution, the differences between the two models are negligible if maximal value of *a* can be accurately deduced. This suggests that the constant OCR model is a suitable model for this geometry regardless of whether the OCR is governed by hyperbolic kinetics or not.

In cylindrical geometry, differences between the constant and hyperbolic consumption rate models were small but still more pronounced than in the spherical case. It is important to note that differences between the two models in cylindrical geometry may be considerably less than that presented here as the analytical approximation systematically overestimates the oxygen profile. Hence, it follows that differences between the constant and hyperbolic consumption rate models are even less than the values reported here. More experimental data are required to investigate this further. The models derived in this work for the cylindrical geometry are Krogh-type models, which have been commonly used to model vessel oxygenation and produce results in good agreement with clinical data [6,8,9]. This approach is not by any means the sole method of estimating oxygen diffusion, and other authors have used numerical methods, including Green’s function methods [14] to solve the reaction–diffusion equations for vessel geometry. A drawback of this method is the difficulty in assigning appropriate boundary conditions [29], a difficulty which appears to some extent in all numerical approaches to oxygen modelling. One advantage of the models proposed in this work is that the boundaries for oxygen diffusion can be readily estimated, circumventing this issue and allowing the establishment of explicit relationships between the OCR and the resultant oxygen diffusion distance. In this work, the illustrative examples show the simplest case of vessels with a constant value for *p*_*o*_ along the length. One limitation of the cylindrical model is that is does not take blood flow or number of erythrocytes in the capillary into account; this could have a significant effect, as the oxygen partial pressure may decrease markedly along a vessel length. If the variation rate of oxygen pressure along the vessel length can be estimated with the relevant perfusion parameters, then it should be possible to extend the simple model presented here to consider this factor.

To gauge sensitivity to the parameter *K*_*m*_, simulations were run from zero to an order of magnitude above literature values (0≤*K*_*m*_≤10 mm Hg). Values of *K*_*m*_ far above literature values greatly increased the diffusion differences between both geometries, and increased the overall oxygen profile significantly. As *K*_*m*_ tended to zero, hyperbolic consumption rate models in both geometries reduced to constant consumption models. As literature values are typically *K*_*m*_≤1 mm Hg for oxygen, the effects of higher values can likely be neglected.

**Figure 6.**
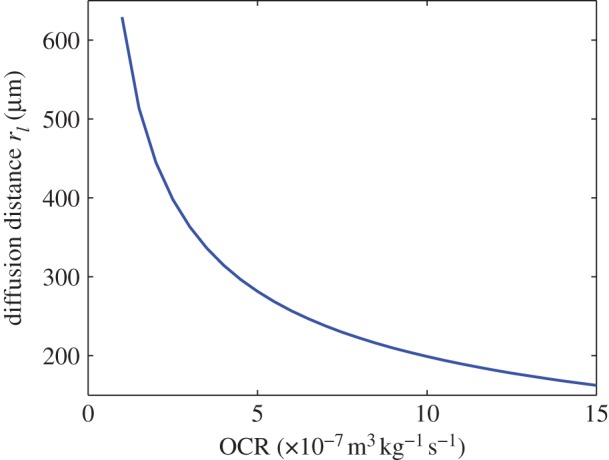
The variation of diffusion limit *r*_*l*_ with OCR *a*_*o*_, illustrated for a spheroid with constant OCR as detailed in equation (2.3).

Whether constant OCR or the hyperbolic approximation models are used, the effect of *a*_*o*_ on oxygen diffusion distance is important and worthy of discussion. Intuitively, we might expect that higher values of maximum consumption rate *a*_*o*_ mean that oxygen is depleted rapidly and as a consequence the oxygen diffusion distance is diminished. The simulations in this work reiterate this in both geometries; in the spherical configuration, increasing *a*_*o*_ corresponded to decreasing values for the viable spherical rim *r*_*c*_ and increased values for the hypoxic radius *r*_*n*_. In cylindrical vessel-like geometry, increasing *a*_*o*_ acted to reduce the diffusion distance *r*_*l*_, as illustrated in figure 6. Unlike many numerical approaches, the relationship between *a*_*o*_ and diffusion distance is explicitly derived in this work. This analysis suggests that OCR has a marked effect on tumour hypoxia in both vascular and avascular situations—for the former, high values of *a*_*o*_ would act to reduce the distance oxygen can travel in the tissue, potentially increasing the prevalence of hypoxic regions. In the avascular case, high values of *a*_*o*_ would result in increased central anoxia. While beyond the scope of this work, this aspect is worthy of further investigation.

The results of this analysis would suggest that in general, constant consumption rate type models yield a broadly similar oxygen profile to hyperbolic-type models and are relatively easy to implement, even for large datasets. There are however situations where significant differences can develop between both approaches, and the forms derived here allow rapid quantification of the resultant oxygen profiles and diffusion distances for either form of reaction term used. Although broadly similar for most realistic parameter values, there are some inherent differences between constant oxygen consumption models and hyperbolic consumption forms, chiefly that the hyperbolic case always results in a deeper diffusion depth than for a constant consumption model with the same parameters. This difference is intuitively grasped by the consideration that OCR falls with decreasing oxygen tension in such models and as a result oxygen can penetrate further at low oxygen levels than in a comparable constant consumption case. This analysis suggests that such differences are usually minor and not typically resolvable through clinical means, but also that there are situations where a measurable difference between both approaches could arise. In the formulation established in this study, the explicit relationship between maximal OCR *a* and the diffusion distances in all cases can be estimated from first principles, allowing rapid quantification of oxygen boundaries in all cases.

## 6. Conclusion

This analysis suggests that since the effective *K*_*m*_ constant for oxygen consumption is so small, only minor differences in diffusion limit (≃10 μm) and local oxygen partial pressure (Δ*p*≤1 mm Hg in spherical geometry, Δ*p*≃2 mm Hg in cylindrical geometry) would be expected between the constant and hyperbolic consumption rate models for the typical range of literature values of *K*_*m*_. There are also cases where differences between both forms can be significant, and the forms outlined in this work allow rapid first principles estimate of the respective oxygen profiles and diffusion distance for both constant consumption rate and hyperbolic consumption rate model forms in spherical and cylindrical geometry.

In general, the simpler constant consumption form can be used in both spherical and cylindrical geometries for most situations. The models outlined also allow quick identification of situations where both models are expected to produce significantly different results, and suggestions even in these limited situations the oxygen profiles for both approaches are broadly similar. In oxygen modelling situations where hyperbolic kinetics are required, the analytical steady-state approximations outlined in this work could be used readily, allowing rapid estimation of the oxygen diffusion limit and projected oxygen profile.

## Supplementary Material

Full solution of equation (13) in Mathematica Format

## Data Accessibility

The full solution of equation (2.12) can be found in Mathematica Format uploaded as supplementary material.
