# Supplementary material for: Oxygen consumption dynamics in steady-state tumour models
Source: R Soc Open Sci. 2014 Sep 24;1(1):140080. doi: 10.1098/rsos.140080 (PMC4448765; doi:10.1098/rsos.140080)
Supplement: Full solution of equation (13) in Mathematica Format [file rsos140080supp1.pdf]

```
(*This Mathematica Notebook yields the analytical solution to Eq. 13 as outlined in the main text. As symbols are as in text unless otherwise specified here. For further information, consult text or davidrobert.grimes@oncology.ox.ac.uk for more information!*)

(*In all ensuing code, A is a constant used for simplicity given by A = (a*omega)/(2D)*)

(*Basic solution (no assumptions)*)

t = DSolve[{p'[r] == A*((r - (rc^2)/r) - ((l)*Log[((rc - r)^2 + l)/l])/r + (2*Sqrt[l]*rc*ArcTan[(rc - r)/Sqrt[l]])/r), p[rc] == 0}, p[r], r]
```

$$\Big\{\Big\{p[r] \to$$

$$\frac{1}{12}\Big(6 A r^2 + i A \sqrt{l}\, \pi^2\, rc - 6 A\, rc^2 - 12 i A \sqrt{l}\, \pi\, rc\, \mathrm{ArcTan}\left[\frac{r-rc}{\sqrt{l}}\right] + 24 i A \sqrt{l}\, rc\, \mathrm{ArcTan}\left[\frac{r-rc}{\sqrt{l}}\right]^2 + 24 i A \sqrt{l}\, rc\, \mathrm{ArcTan}\left[\frac{r-rc}{\sqrt{l}}\right] \mathrm{ArcTan}\left[\frac{rc}{\sqrt{l}}\right] + 12 A \sqrt{l}\, \pi\, rc\, \mathrm{Log}[2] - 12 A \sqrt{l}\, \pi\, rc\, \mathrm{Log}\left[1 + e^{-2 i\, \mathrm{ArcTan}\left[\frac{r-rc}{\sqrt{l}}\right]}\right] + 24 A \sqrt{l}\, rc\, \mathrm{ArcTan}\left[\frac{r-rc}{\sqrt{l}}\right] \mathrm{Log}\left[1 + e^{-2 i\, \mathrm{ArcTan}\left[\frac{r-rc}{\sqrt{l}}\right]}\right] + 24$$

$$A \sqrt{l}\, rc\, \mathrm{ArcTan}\left[\frac{rc}{\sqrt{l}}\right] \mathrm{Log}\left[1 - e^{2 i\, \mathrm{ArcTan}\left[\frac{rc}{\sqrt{l}}\right]}\right] - 24 A \sqrt{l}\, rc\, \mathrm{ArcTan}\left[\frac{r-rc}{\sqrt{l}}\right] \mathrm{Log}\left[1 - e^{2 i \left(\mathrm{ArcTan}\left[\frac{r-rc}{\sqrt{l}}\right] + \mathrm{ArcTan}\left[\frac{rc}{\sqrt{l}}\right]\right)}\right] - 24 A \sqrt{l}\, rc\, \mathrm{ArcTan}\left[\frac{rc}{\sqrt{l}}\right] \mathrm{Log}\left[1 - e^{2 i \left(\mathrm{ArcTan}\left[\frac{r-rc}{\sqrt{l}}\right] + \mathrm{ArcTan}\left[\frac{rc}{\sqrt{l}}\right]\right)}\right] - 12 A\, rc^2\, \mathrm{Log}[r] + 12 A \sqrt{l}\, \pi\, rc\, \mathrm{Log}\left[\frac{1}{\sqrt{\frac{l+(r-rc)^2}{l}}}\right] - 12 A\, l\, \mathrm{Log}[r]\, \mathrm{Log}\left[1 + \frac{(r-rc)^2}{l}\right] +$$

$$12 A\, l\, \mathrm{Log}[r]\, \mathrm{Log}\left[-i\sqrt{l} + r - rc\right] + 12 A\, l\, \mathrm{Log}[r]\, \mathrm{Log}\left[i\sqrt{l} + r - rc\right] - 12 A\, l\, \mathrm{Log}\left[-i\sqrt{l} + r - rc\right] \mathrm{Log}\left[-\frac{i r}{\sqrt{l} - i\, rc}\right] - 12 A\, l\, \mathrm{Log}\left[i\sqrt{l} + r - rc\right] \mathrm{Log}\left[\frac{i r}{\sqrt{l} + i\, rc}\right] + 12 A\, rc^2\, \mathrm{Log}[rc] - 12 A\, l\, \mathrm{Log}\left[-i\sqrt{l}\right] \mathrm{Log}[rc] - 12 A\, l\, \mathrm{Log}\left[i\sqrt{l}\right] \mathrm{Log}[rc] +$$

$$12 A\, l\, \mathrm{Log}\left[-i\sqrt{l}\right] \mathrm{Log}\left[-\frac{i\, rc}{\sqrt{l} - i\, rc}\right] + 12 A\, l\, \mathrm{Log}\left[i\sqrt{l}\right] \mathrm{Log}\left[\frac{i\, rc}{\sqrt{l} + i\, rc}\right] - 24 A \sqrt{l}\, rc\, \mathrm{ArcTan}\left[\frac{rc}{\sqrt{l}}\right] \mathrm{Log}\left[\frac{rc}{\sqrt{l}\sqrt{1 + \frac{rc^2}{l}}}\right] + 24 A \sqrt{l}\, rc\, \mathrm{ArcTan}\left[\frac{rc}{\sqrt{l}}\right] \mathrm{Log}\left[\mathrm{Sin}\left[\mathrm{ArcTan}\left[\frac{r-rc}{\sqrt{l}}\right] + \mathrm{ArcTan}\left[\frac{rc}{\sqrt{l}}\right]\right]\right] + 12 i A \sqrt{l}\, rc\, \mathrm{PolyLog}\left[2, -e^{-2 i\, \mathrm{ArcTan}\left[\frac{r-rc}{\sqrt{l}}\right]}\right] -$$

$$12 i A \sqrt{l}\, rc\, \mathrm{PolyLog}\left[2, e^{2 i\, \mathrm{ArcTan}\left[\frac{rc}{\sqrt{l}}\right]}\right] + 12 i A \sqrt{l}\, rc\, \mathrm{PolyLog}\left[2, e^{2 i \left(\mathrm{ArcTan}\left[\frac{r-rc}{\sqrt{l}}\right] + \mathrm{ArcTan}\left[\frac{rc}{\sqrt{l}}\right]\right)}\right] + 12 A\, l\, \mathrm{PolyLog}\left[2, \frac{\sqrt{l}}{\sqrt{l} - i\, rc}\right] - 12 A\, l\, \mathrm{PolyLog}\left[2, \frac{\sqrt{l} + i(r - rc)}{\sqrt{l} - i\, rc}\right] + 12 A\, l\, \mathrm{PolyLog}\left[2, \frac{\sqrt{l}}{\sqrt{l} + i\, rc}\right] - 12 A\, l\, \mathrm{PolyLog}\left[2, \frac{\sqrt{l} - i r + i\, rc}{\sqrt{l} + i\, rc}\right]\Big)\Big\}\Big\}$$

```
(*Simplifying assumptions (domains of validity) *)

u = FullSimplify[t, rc > 0 && r > 0 && rc >= r && l > 0 && A > 0 && p ≥ 0]
```

$$\Big\{\Big\{p[r] \to \frac{1}{12} A \Big(6\, l\, \pi^2 + i\sqrt{l}\, \pi^2\, rc + 6 (r - rc)(r + rc) + 6 \Big(4 i \sqrt{l}\, rc\, \mathrm{ArcTan}\left[\frac{r-rc}{\sqrt{l}}\right]^2 + 2\sqrt{l}\, rc\, \mathrm{ArcTan}\left[\frac{r-rc}{\sqrt{l}}\right] \Big(-i\pi + 2 i\, \mathrm{ArcTan}\left[\frac{rc}{\sqrt{l}}\right] + \mathrm{Log}[4] - 2\,\mathrm{Log}\left[1 - e^{2 i \left(\mathrm{ArcTan}\left[\frac{r-rc}{\sqrt{l}}\right] + \mathrm{ArcTan}\left[\frac{rc}{\sqrt{l}}\right]\right)}\right] + \mathrm{Log}[l] - 2\,\mathrm{Log}\left[\sqrt{l} + i(r - rc)\right]\Big) + 2\sqrt{l}\, \pi\, rc\, \mathrm{Log}\left[\sqrt{l} + i(r - rc)\right] -$$

$$\sqrt{l}\, \pi\, rc\, \mathrm{Log}\left[l + (r - rc)^2\right] - 2\, l\, \mathrm{Log}[r]\, \mathrm{Log}\left[1 + \frac{(r-rc)^2}{l}\right] + 2\, rc^2\, \mathrm{Log}\left[\frac{rc}{r}\right] - l\, \mathrm{Log}[l]\, \mathrm{Log}\left[-i\sqrt{l} + rc\right] + 2\, l\, \mathrm{Log}\left[i\sqrt{l} + r - rc\right] \mathrm{Log}\left[-i\sqrt{l} + rc\right] - i\, l\, \pi\, \mathrm{Log}\left[l\left(-i\sqrt{l} + rc\right)\right] + i\, l\, \pi\, \mathrm{Log}\left[-\frac{1}{i\sqrt{l} + rc}\right] +$$

$$l\, \mathrm{Log}[l]\, \mathrm{Log}\left[-\frac{1}{i\sqrt{l} + rc}\right] + 2 i\, l \left(\pi + i\, \mathrm{Log}\left[-\frac{1}{i\sqrt{l} + rc}\right]\right) \mathrm{Log}\left[i\sqrt{l} - r + rc\right] + 4\sqrt{l}\, rc\, \mathrm{ArcTan}\left[\frac{rc}{\sqrt{l}}\right] \left(-i\pi + \mathrm{Log}\left[\frac{2 i\, rc}{\sqrt{l} - i\, rc}\right] - \mathrm{Log}\left[-\frac{2 i\, r\, rc \sqrt{\frac{l}{l + rc^2}}}{l - i\sqrt{l}\, r - r\, rc + rc^2}\right] + \mathrm{Log}\left[\mathrm{Sin}\left[\mathrm{ArcTan}\left[\frac{r-rc}{\sqrt{l}}\right] + \mathrm{ArcTan}\left[\frac{rc}{\sqrt{l}}\right]\right]\right]\right)\Big)\Big) +$$

$$i A \sqrt{l}\, rc \left(\mathrm{PolyLog}\left[2, -e^{-2 i\, \mathrm{ArcTan}\left[\frac{r-rc}{\sqrt{l}}\right]}\right] - \mathrm{PolyLog}\left[2, e^{2 i\, \mathrm{ArcTan}\left[\frac{rc}{\sqrt{l}}\right]}\right] + \mathrm{PolyLog}\left[2, e^{2 i \left(\mathrm{ArcTan}\left[\frac{r-rc}{\sqrt{l}}\right] + \mathrm{ArcTan}\left[\frac{rc}{\sqrt{l}}\right]\right)}\right]\right) + A\, l\, \mathrm{PolyLog}\left[2, \frac{1}{1 - \frac{i\, rc}{\sqrt{l}}}\right] + A\, l\, \mathrm{PolyLog}\left[2, \frac{1}{1 + \frac{i\, rc}{\sqrt{l}}}\right] - A\, l\, \mathrm{PolyLog}\left[2, 1 - \frac{r}{-i\sqrt{l} + rc}\right] -$$

$$A\, l\, \mathrm{PolyLog}\left[2, 1 - \frac{r}{i\sqrt{l} + rc}\right]\Big\}\Big\}$$

```
(*Further refinement*)

SolutionForm = refine[u]
```

$$\mathrm{refine}\Big[$$

$$\Big\{\Big\{p[r] \to \frac{1}{12} A \Big(6\, l\, \pi^2 + i\sqrt{l}\, \pi^2\, rc + 6 (r - rc)(r + rc) + 6 \Big(4 i \sqrt{l}\, rc\, \mathrm{ArcTan}\left[\frac{r-rc}{\sqrt{l}}\right]^2 + 2\sqrt{l}\, rc\, \mathrm{ArcTan}\left[\frac{r-rc}{\sqrt{l}}\right] \Big(-i\pi + 2 i\, \mathrm{ArcTan}\left[\frac{rc}{\sqrt{l}}\right] + \mathrm{Log}[4] - 2\,\mathrm{Log}\left[1 - e^{2 i \left(\mathrm{ArcTan}\left[\frac{r-rc}{\sqrt{l}}\right] + \mathrm{ArcTan}\left[\frac{rc}{\sqrt{l}}\right]\right)}\right] + \mathrm{Log}[l] - 2\,\mathrm{Log}\left[\sqrt{l} + i(r - rc)\right]\Big) + 2\sqrt{l}\, \pi\, rc\, \mathrm{Log}\left[\sqrt{l} + i(r - rc)\right] - \sqrt{l}$$

$$\pi\, rc\, \mathrm{Log}\left[l + (r - rc)^2\right] - 2\, l\, \mathrm{Log}[r]\, \mathrm{Log}\left[1 + \frac{(r-rc)^2}{l}\right] + 2\, rc^2\, \mathrm{Log}\left[\frac{rc}{r}\right] - l\, \mathrm{Log}[l]\, \mathrm{Log}\left[-i\sqrt{l} + rc\right] + 2\, l\, \mathrm{Log}\left[i\sqrt{l} + r - rc\right] \mathrm{Log}\left[-i\sqrt{l} + rc\right] - i\, l\, \pi\, \mathrm{Log}\left[l\left(-i\sqrt{l} + rc\right)\right] + i\, l\, \pi\, \mathrm{Log}\left[-\frac{1}{i\sqrt{l} + rc}\right] +$$

$$l\, \mathrm{Log}[l]\, \mathrm{Log}\left[-\frac{1}{i\sqrt{l} + rc}\right] + 2 i\, l \left(\pi + i\, \mathrm{Log}\left[-\frac{1}{i\sqrt{l} + rc}\right]\right) \mathrm{Log}\left[i\sqrt{l} - r + rc\right] + 4\sqrt{l}\, rc\, \mathrm{ArcTan}\left[\frac{rc}{\sqrt{l}}\right] \left(-i\pi + \mathrm{Log}\left[\frac{2 i\, rc}{\sqrt{l} - i\, rc}\right] - \mathrm{Log}\left[-\frac{2 i\, r\, rc \sqrt{\frac{l}{l + rc^2}}}{l - i\sqrt{l}\, r - r\, rc + rc^2}\right] + \mathrm{Log}\left[\mathrm{Sin}\left[\mathrm{ArcTan}\left[\frac{r-rc}{\sqrt{l}}\right] + \mathrm{ArcTan}\left[\frac{rc}{\sqrt{l}}\right]\right]\right]\right)\Big)\Big) +$$

$$i A \sqrt{l}\, rc \left(\mathrm{PolyLog}\left[2, -e^{-2 i\, \mathrm{ArcTan}\left[\frac{r-rc}{\sqrt{l}}\right]}\right] - \mathrm{PolyLog}\left[2, e^{2 i\, \mathrm{ArcTan}\left[\frac{rc}{\sqrt{l}}\right]}\right] + \mathrm{PolyLog}\left[2, e^{2 i \left(\mathrm{ArcTan}\left[\frac{r-rc}{\sqrt{l}}\right] + \mathrm{ArcTan}\left[\frac{rc}{\sqrt{l}}\right]\right)}\right]\right) + A\, l\, \mathrm{PolyLog}\left[2, \frac{1}{1 - \frac{i\, rc}{\sqrt{l}}}\right] + A\, l\, \mathrm{PolyLog}\left[2, \frac{1}{1 + \frac{i\, rc}{\sqrt{l}}}\right] - A\, l\, \mathrm{PolyLog}\left[2, 1 - \frac{r}{-i\sqrt{l} + rc}\right] - A\, l\, \mathrm{PolyLog}\left[2, 1 - \frac{r}{i\sqrt{l} + rc}\right]\Big\}\Big\}\Big]$$
